# Matching Development of Point-of-Care Diagnostic Tests to the Local Context: A Case Study of Visceral Leishmaniasis in Kenya and Uganda

**DOI:** 10.9745/GHSP-D-20-00028

**Published:** 2020-09-30

**Authors:** Michel Bengtson, Mitasha Bharadwaj, Astrid ten Bosch, Hellen Nyakundi, Damaris Matoke-Muhia, Cees Dekker, Jan-Carel Diehl

**Affiliations:** a Department of Bionanoscience, Kavli Institute of Nanoscience Delft, Delft University of Technology, The Netherlands.; b Department of Sustainable Design Engineering, Section of Design for Sustainability, Faculty of Industrial Design Engineering, Delft University of Technology, The Netherlands.; c University of Nairobi, Kenya.; d Centre for Biotechnology Research and Development, Kenya Medical Research Institute, Kenya.

## Abstract

The rapid growth of point-of-care (POC) diagnostic tests necessitates a clear vision of when, where, and why a new POC diagnostic test needs to be developed and how it can be used in a way that matches a local health care context. Here, we present an innovative approach toward developing a concept target product profile (CTPP), which is a new mapping tool that helps researchers match a new diagnostic test to a specific local health care context early in the research and development process. As a case study, we focus on the diagnosis of visceral leishmaniasis (VL) in rural resource-limited regions of Kenya and Uganda. Our stepwise approach integrates elements of design thinking and uses a combination of literature reviews and field research for a context analysis of local health care systems and practices. We then use visual thinking in the form of Gigamaps and patient journeys to identify use case scenarios and to present our findings from the field research to key stakeholders. The use case scenarios describe the diagnostic scope of a new POC test based on the feasibility of the new test, the local need, and the contextual fit. For our case study of VL, we identify 2 valuable use case scenarios, namely test-of-cure and screening and confirmation, and we formulate a CTPP. We anticipate that a CTPP will enable researchers to match a new POC diagnostic test during the research and development process to the local health care context in which it will be used.

## Abstract (precis)

We provide a new protocol to connect how findings from field research on the local health care setting in resource-limited regions can inform researchers that are working toward developing a new point-of-care diagnostic test for neglected tropical diseases.

## Teaser

**Key Findings:**

- A concept target product profile is an effectivenew tool that can aid researchers to develop a technology to match specific health care context more quickly than a conventional target product profile.
- The role of local volunteers and community health care workers is critically important for access to diagnostics in resource-limited settings.
- A noninvasive test-of-cure and a screening and confirmation test will significantly improve management of visceral leishmaniasis in endemic regions.

**Key Implications:**

- Early during the research and development stage, researchers should consider who will administer the test (patient, health care worker, doctor) and for what purpose.
- Program managers should consider that the training level of staff and volunteers and the availability of the resources are the critical determinants for the use of a diagnostic test.

## INTRODUCTION

Within the past decade, point-of-care (POC) diagnostic tests have received immense attention^1^ because their accuracy and ease of use create the ideal solution for early diagnostics of infectious diseases in resource-limited settings. POC diagnostic tests should involve a minimum number of steps to obtain a real-time result that is easy to interpret. Importantly, POC tests are designed to be performed near or at the site where the patient is to enable a short turnaround time for them to receive treatment and care.^2^ Given these favorable characteristics, novel POC technologies for decentralized diagnostics are being developed worldwide at a rapid rate.^3^ Ample research opportunities exist for continued development of POC diagnostic tests, particularly for neglected tropical diseases (NTDs), which are a group of chronic, disabling, and potentially fatal diseases that are prevalent in tropical and subtropical regions.^4^ NTDs occur predominantly in resource-limited settings, which are defined by the World Bank as resource-constrained (human, environmental, economical) regions with limited infrastructure and/or basic services in low- or middle-income countries.^5^ By definition, NTDs receive little attention, and 14 of the 24 NTDs that are currently acknowledged by the World Health Organization (WHO) lack essential POC diagnostic tests.^6^

It is crucial to ensure that new POC diagnostic tests meet the needs of the (multiple) end users and fit constraints within local health care contexts.^7^^,^^8^ Particularly for resource-limited settings, innovative approaches are required to meet the demands for diagnostics that are suitable for use at the lowest and most constrained level of the health care system. Current POC diagnostic tests are often not compatible with the most constrained level of the health care systems in resource-limited settings because they still require resources, such as a cold chain to store reagents or trained users who are generally not available in such settings. Although these resources are available at higher levels of the health care system (reference laboratories and hospitals), they are not available in the local clinics in remote areas where patients first seek health care. This situation necessitates new POC diagnostic tests that do not depend on additional resources and can be administered by users without extensive training.

Multiple guidelines are available that can be used to jointly guide the design of new POC diagnostic tests that address the needs of the end users and stakeholders in a local health care context. WHO developed the ASSURED criteria (i.e., a diagnostic test should be affordable, sensitive, specific, user-friendly, rapid and robust, equipment-free, and deliverable to end users) to guide the development of new medical technologies and to encourage the adaptation of existing technologies to suit resource-limited settings better. However, the ASSURED criteria are rather general and broadly applicable.^9^ WHO and other organizations, such as the Drugs for Neglected Diseases initiative and the Foundation for Innovative New Diagnostics, collaborate with various stakeholders and experts to develop target product profiles (TPPs), which are more specific guidelines that provide details on the minimal and optimal performance and operational features of diagnostic tests. TPPs are the end result of several rounds of discussions to reach a consensus from policy makers,^10^ which is an important yet labor-intensive process. TPPs are developed when the use cases are already defined and it is known when, where, and why the test will be used,^11^^,^^12^ that is, when specifications have been determined regarding the diagnostic moment (when the patient gets tested), the diagnostic setting (where the patient gets tested), and the purpose of the test (why the patient gets tested), which could be screening and confirmation.

While developing a novel POC diagnostic test, researchers would benefit from having guidelines that are less abstract than the ASSURED criteria, which may be too broad to be effective for a specific context and disease but are less involved and less prescriptive than a full TPP. Indeed, a POC diagnostic test that does not meet all the stringent requirements of a TPP can still be of great value, depending on the local health care context and the needs of the end users.^13^ For example, if a TPP defines a desired sensitivity of a test, but a new test is somewhat less sensitive (and thus would be excluded by the TPP) yet is much more stable at higher temperatures or an order of magnitude cheaper, it could still be valuable for a specific disease in an endemic tropical region.^13^ To facilitate the design of a POC diagnostic test that fits a health care context, we conclude that an efficient and effective mapping tool is needed, particularly when the diagnostic moment, setting, and purpose of the test are not yet defined.

**FIGURE 1.**
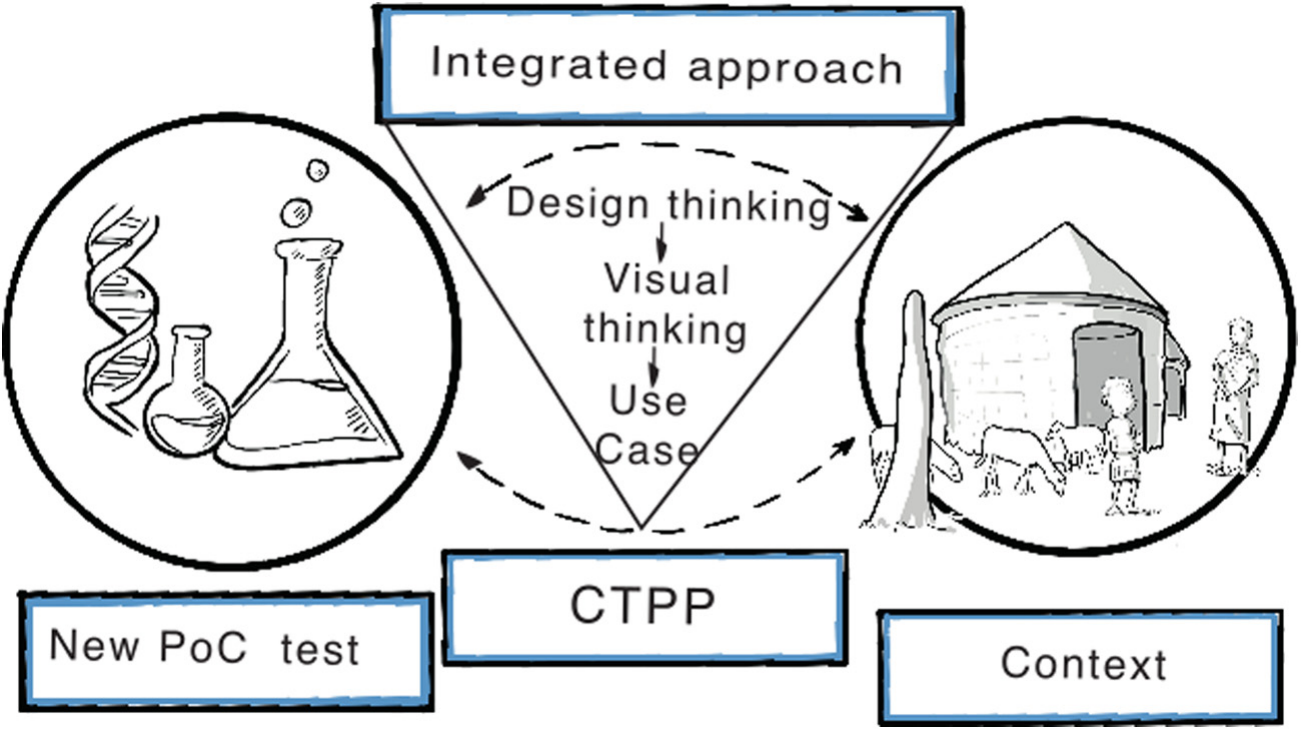
Schematic Representation of a CTPP That Matches the Gap Between a New POC Test and the Local Health Care Context Abbreviations: CTPP, concept target product profile; PoC, point-of-care.

To address this need, we propose to formulate a concept target product profile (CTPP) as an intermediate guideline for developing a diagnostic test that addresses the needs of the end users in the local health care context. A CTPP does not replace a TPP, and it best serves as an intermediate guideline for researchers in the form of a mapping tool. Such a CTPP should preferably be developed at the onset of the research and development (R&D) of a new POC diagnostic test, aiding researchers in considering the context at an early stage while the POC diagnostic test is designed and developed. A CTPP would enable researchers to identify the minimum features for successful implementation of a POC diagnostic test in a local health care context, and it will cost considerably less time and resources than the development of a TPP. To illustrate, a recent TPP for dermal leishmaniases took approximately 4 years and 82 experts to reach a consensus,^7^ whereas a CTPP as proposed in this study can take 6 months and a considerably smaller team depending on the interdisciplinary team of choice. Although a CTPP will serve a clear purpose, a full TPP is still required because it is extremely valuable for experts such as policymakers and provides guidelines for further test development by technical experts. In this paper, we propose a step-by-step approach, which includes elements of design thinking,^14^ toward developing a CTPP that matches the new POC diagnostic test and the local health care context in which it will be used (Figure 1).

A concept target product profile best serves as an intermediate mapping tool for researchers to use to develop a diagnostic test that addresses the needs of end users in local health care contexts.

## METHODOLOGY

For the development of a CTTP, we adopted a stepwise approach, using design-thinking principles (Box). Design thinking is a human-centered approach to innovation that integrates the needs of people (desirability), the possibilities of the technology (feasibility), and the requirements for business success (viability). Design thinking encourages novel and thorough solutions. In general, design thinking is utilized to evaluate the current situation, to identify the actual problem, and thereby provide a guideline for developing customized solutions. Core principles of design thinking are to empathize, define, ideate, prototype, and test.^15^ The first 3 of these principles have been integrated into our stepwise approach for formulating a CTPP.

BOX. Design-Thinking Principles

Design thinking refers to a strategic process in which design concepts are developed (e.g., proposal for a new diagnostic test) before final product development. It is essentially an iterative method for problem solving. The use of design thinking encompasses the innovation of new products within social and business contexts. In this way, designers actively seek to understand the user and their experience before they try to solve the problem and move into the product development process. Therefore, design thinking involves methods such as context analysis, problem framing, creating thinking, prototyping, testing, and (re)evaluating.

Core principles of design thinking are to empathize, define, ideate, prototype, and test. Design thinking aims to create a solution that is desirable (for the end user and stakeholders), (technically) feasible, and (economically) viable. Within the broad context of design thinking, tools such as visual thinking can be used to facilitate the design-thinking process. Visual thinking allows people from diverse backgrounds to share insights, synthesize existing information, and formulate superior ideas. Visual thinking tools enable designers to convey complex ideas to the stakeholders in a logical manner. To summarize, design thinking is an unconventional toolbox that within its visual thinking framework can facilitate the communication of complex problems and thereby aid in inventing/devising novel solutions to complex problems.

In our case, the design-thinking principles were used to evaluate current diagnostic practices for a disease, identify the diagnostic need for a disease, and provide a diagnostic solution. The approach started with a comprehensive literature survey, followed by observations and semistructured interviews with the health care providers in the field and scientific researchers. In this step, the design principle empathize was integrated to gain an empathetic understanding of the problem at hand. Thereafter, the design-thinking principle define was utilized to combine the information gathered and identify the problem at hand. This step used a set of design tools known as “visual thinking.”^16^^–^^18^ Specifically, “Gigamaps” were used to describe the health care system and define the current diagnostic need. Gigamaps are large and information-dense diagrams that act as a bridge between inquiry, design, and implementation.^18^ Using these, researchers are intentionally encouraged to identify and subsequently use patterns that emerge from the field observations and data.^19^ Thereafter, visual depictions of the patient journeys were constructed based on the information gathered from literature and field research. The term patient journey refers to the experiences and processes that a patient goes through during the course of a disease and its treatment.^20^^,^^21^ These patient journeys provide a detailed yet simplified overview of the challenges faced by the patients while seeking effective diagnoses and subsequent treatment. Finally, the design-thinking principle ideate was applied to obtain the logical solution for the diagnostic needs defined in the previous step. Again, a visual thinking tool in the form of use cases, referred to as scenarios, was utilized to present the complex problem and logical solution. Based on the outcomes from our approach, a CTPP was formulated that integrated a desirable, feasible, and viable solution to a complex societal problem.

The above methodology is expected to be broadly applicable in the field of diagnostics for use in endemic resource-limited settings, since context analysis is always essential to guide the design process during R&D. Developing a CTPP requires the following seven steps.

A CTPP is expected to be broadly applicable for diagnostics in endemic resource-limited settings.

### Step 1: Literature Review

A critical review of the existing literature on a disease and its relevant health care context is performed to gauge the disease endemicity, resource availability, and current diagnostic practices in the resource-constrained settings. Such critical analyses help in identifying the potential and the limitations of the existing diagnostic practices and in identifying stakeholders such as patients, health care staff, patient families, health care organizations, nongovernmental organizations, and government bodies, and thereby highlight the implementation needs for novel diagnostic solutions.

### Step 2: Selection of an Endemic Resource-Limited Region for a Case Study

Beyond mere literature study, it is important to direct field research for a case study. Selection criteria for a fitting case should include (i) an endemic region that bears a high burden of a disease; (ii) an area that has an urgent need for POC diagnostic tests; and (iii) a politically stable conflict-free zone for safety and logistic capacity (roads and access to remote endemic areas) for field research.

### Step 3: Field Research With Direct Observations and Interviews With Stakeholders

Despite an extensive literature survey, many aspects of diagnoses in remote endemic regions often remain unclear, such as the clinical algorithm for disease identification, the patient’s journey from becoming infected to getting treatment, the availability of laboratory equipment, and the level of trained personnel at the lowest most constrained level of the health care system. To obtain observations from the field for filling the knowledge gaps in the literature, a field trip to the selected endemic regions was necessary. The objective of such a field trip is to gather direct observations at various health care levels in that region and to carry out semistructured interviews in the field to obtain expert input from key stakeholders as identified from the literature survey and from advice of the locals.

### Step 4: Create Gigamaps and Patient Journeys Based on Insights Gathered

To get a deeper understanding of the diagnostic practices for a disease, the assumptions made by the detailed literature survey (step 1) are analyzed in conjunction with the information that was obtained from semistructured interviews with the health care professionals (step 3). The observations are processed and organized to highlight the limitations of existing diagnostic practices. Since the health care context of a disease in resource-limited settings is complex with social and technical challenges and involves a wide range of stakeholders, understanding the corresponding diagnostic practices for a disease can be quite challenging. To deal with the complexity of such a multifaceted health care system, visual thinking tools are used to present the complex diagnostic problem at hand (define) and to identify the logical solution (ideate) in the form of use case scenarios. Visual Gigamaps are created to outline current diagnostic practices at various stages of the disease, and health care-seeking behaviors of patients from a health care provider’s point of view across different health care levels. Next, visual patient journeys are constructed within the selected disease endemic region. In our approach, these extended patient journeys represent a sequence of interactions between the patient, the health care system, and the stakeholders involved, and they consider both the technical, economic, and social factors.

### Step 5: Create Use Case Scenarios

Next, we create various scenarios for the application of a diagnostic test within the selected region. Again, visual thinking is utilized to present every scenario to provide a clear understanding of the requisite diagnostic need and to suggest logical solutions.

### Step 6: Validation of the Use Case Scenarios

Visualizations of the scenarios from step 5 are used to facilitate discussions with different stakeholders with an objective to critically select and thus validate the most valuable scenario through which a diagnostic test can meet the local needs of the end users and stakeholders. Thus, detailed discussions are conducted with the stakeholders on each scenario to obtain the most pressing diagnostic need and to define the priorities from the perspective of health care providers.

### Step 7: Formulation of a CTPP

Finally, the stepwise approach collectively leads to the formulation of a CTPP for a diagnostic test that will be most effective for the selected disease-endemic region. The CTPP is formulated for the most urgent diagnostic need as viewed from the perspective of health care providers.

## RESULTS OF A CASE STUDY: DEVELOPING A CTPP FOR A POC DIAGNOSTIC TEST FOR VL IN KENYA AND UGANDA

For proof of principle, we selected the NTD visceral leishmaniasis (VL) as a case study to validate our CTPP design approach. Below we explicate the above 7 steps for this case study.

### 1. Literature Review on VL Endemicity and VL Health Care Context

Leishmaniases are caused by more than 20 different *Leishmania* species, which are parasites that can be transmitted to humans and other animals by the bite of infected female phlebotomine sand flies.^22^^,^^23^ Worldwide, there are approximately 2 million new cases each year, and 556 million people are at risk of acquiring the infection.^24^ VL (also known as Kala-azar) is the second-largest parasitic killer after malaria^25^^,^^26^ and the most severe form of leishmaniasis because it affects the visceral organs, particularly the liver, spleen, and lymph nodes.^27^ Although VL is curable, it remains a fatal disease because it is often left untreated due to its low index of suspicion by health care providers, late diagnosis, and inadequate case management, especially at an early stage in low-resource settings.^28^ Furthermore, the initial symptoms of VL (e.g., persistent fever, weight loss, fatigue, and anemia) overlap with other febrile illnesses, such as malaria, and hence it is often misdiagnosed and treated incorrectly. If left untreated, VL is fatal within 2 years, due to severe anemia or secondary bacterial infections.^29^ Furthermore, post-kala-azar dermal leishmaniasis (PKDL) is a skin condition that occurs after VL treatment due to persisting parasites in the skin.^29^ Incomplete treatment is a major risk factor for PKDL.^29^ Although PKDL lesions are typically self-healing, they pose as infectious reservoirs for sandflies^29^ and cause aesthetic and psychological complications that affect the patient’s quality of life, especially young adults. VL is a complex disease that is further complicated by coinfections such as HIV.^28^^,^^30^^,^^31^ Individuals with HIV who are immunosuppressed often present with more severe VL symptoms and require different treatment regimens.^31^ Other common coinfections in VL endemic regions include malaria^32^ and tuberculosis.^33^ Thus VL needs to be diagnosed and managed on a case-by-case basis due to confounding conditions such as immunosuppression.^13^

VL is curable but remains a fatal disease because it is often left untreated, especially at early stages in low-resource settings.

**FIGURE 2.**
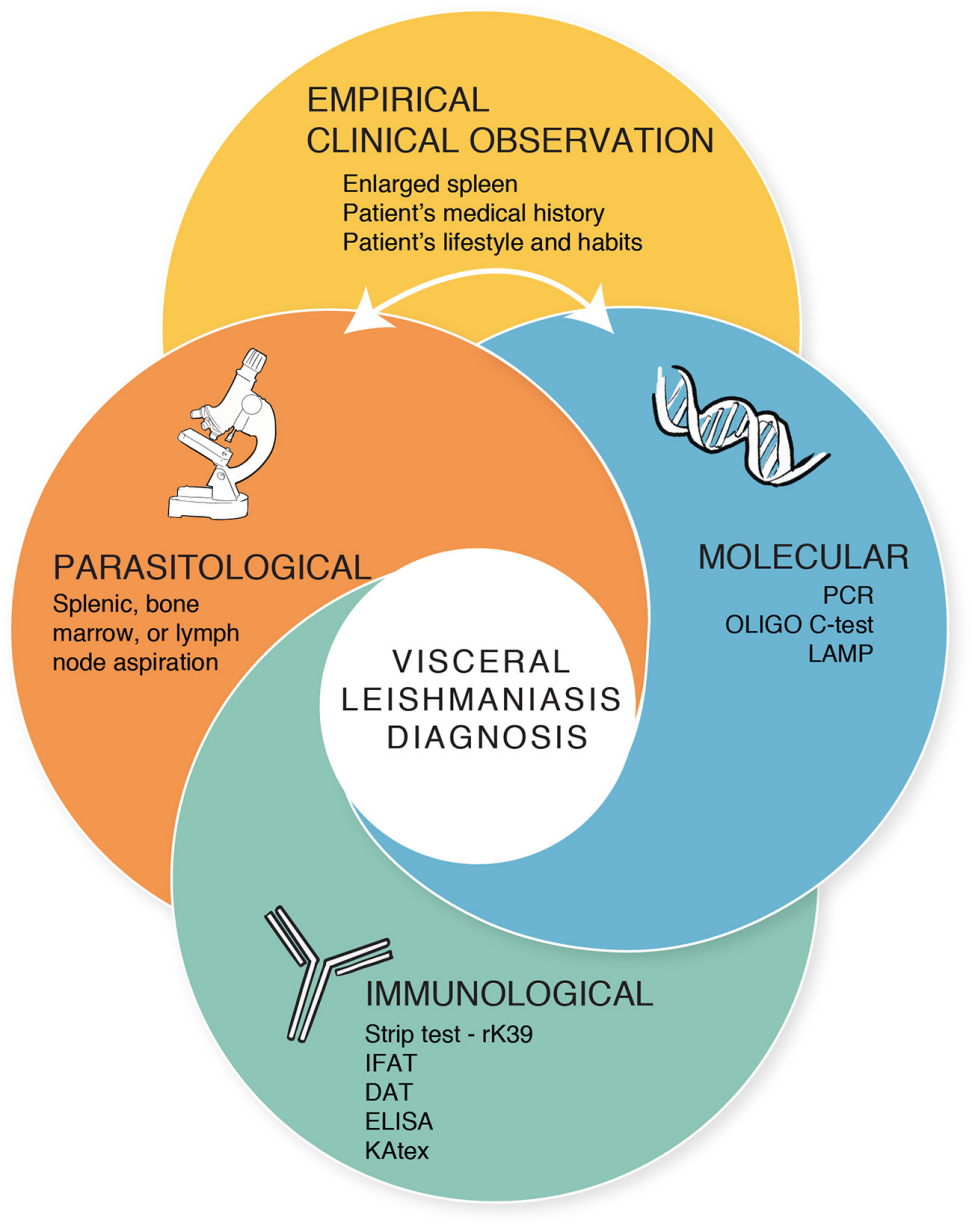
Current Methods Used for Diagnosing Visceral Leishmaniasis^a^ Abbreviations: DAT, direct agglutination test; ELISA, enzyme-linked immunosorbent assay; IFAT, immunofluorescent-antibody test; KAtex, latex agglutination test; LAMP, loop-mediated isothermal amplification; PCR, polymerase chain reaction.^a^ After an empirical clinical observation is done, a screening test (usually, an immunological test) is administered, depending upon the availability.

VL is currently diagnosed by using either one or a combination of the following: (1) empirical clinical observations,^31^ (2) immunological rapid diagnostic tests (RDTs) and/or immunoassays, (3) molecular tests to detect the pathogen’s DNA in clinical samples, and (4) parasitological tests that require microscopic analyses of invasive splenic or bone marrow aspirations^34^^,^^31^ (Figure 2). Except for clinical methods and RDTs, which can be less reliable, these diagnostic practices require expensive instruments, a stable source of electricity, a well-equipped laboratory, and an expert to operate; therefore, they are inadequate for use within resource-limited settings.^30^^,^^35^ The most readily used test for VL diagnosis, especially in remote settings, is the rK39 RDT.^22^ However, since the rK39 RDT is an immunological test, it is less reliable because the sensitivity of the test differs between individuals,^29^^,^^36^ and it cannot serve as a test-of-cure due to persisting antibodies after treatment.^30^^,^^37^

### 2. Select a Region for a Case Study

**FIGURE 3.**
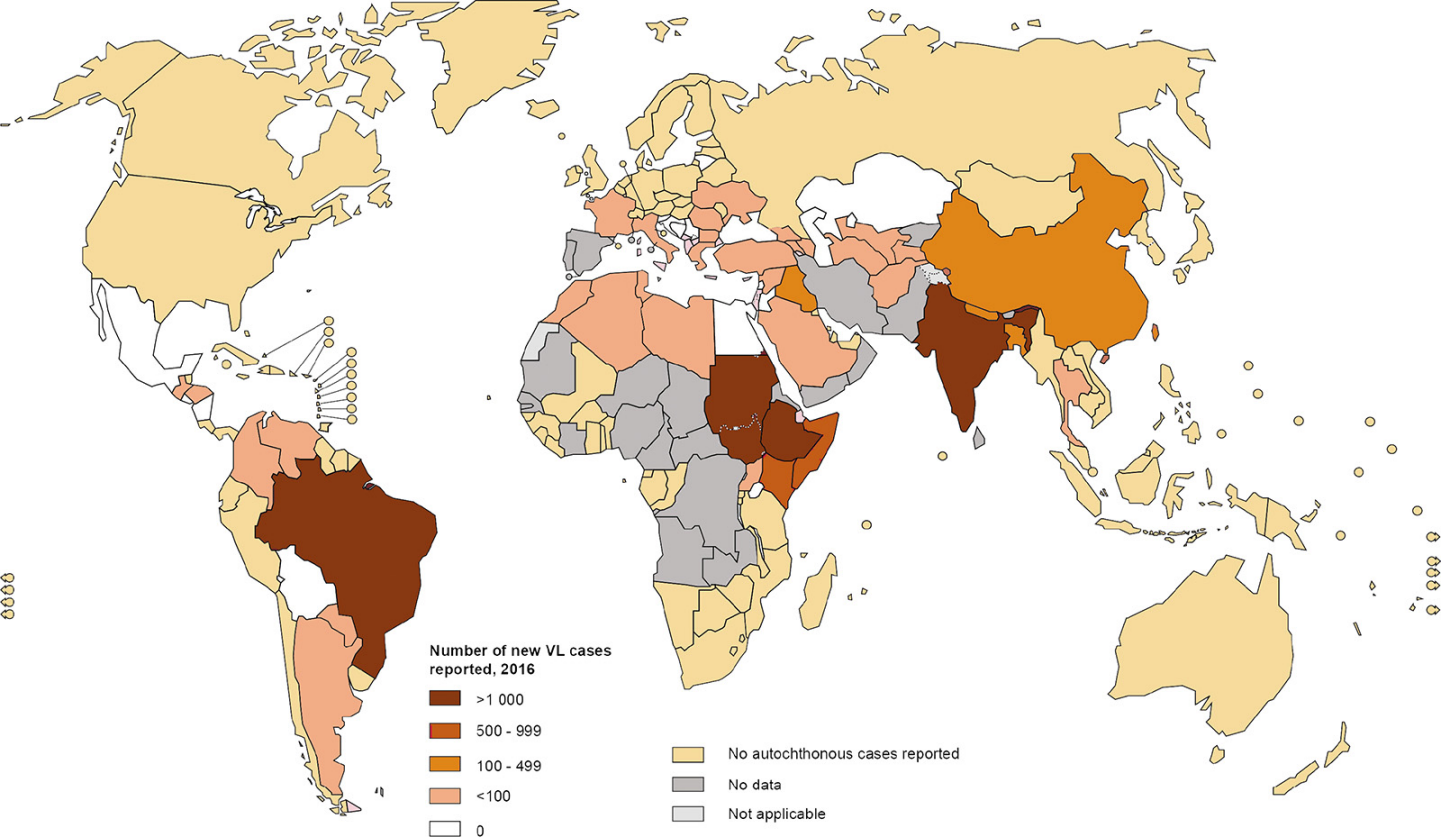
Status of Endemicity of Visceral Leishmaniasis Worldwide^38^

Leishmaniases remain endemic in more than 98 countries with the majority of VL cases in South Asia (India, Nepal and Bangladesh), South America (Brazil), and the horn of Africa (Ethiopia, Somalia, South Sudan, Sudan, Kenya, and Uganda) (Figure 3).^31^^,^^38^ Seven countries in particular (India, Brazil, Ethiopia, Kenya, Somalia, South Sudan, and Sudan), reported approximately 90% of the global cases of VL in 2015.^29^ Although various VL control programs that focus on prevention and treatment are operational within these countries,^22^ the current rK39 RDT has been shown to have a poor performance in East Africa compared with India,^29^^,^^36^ necessitating an improved diagnostic approach in East African countries.

Considering the global spread of VL, our first challenge was to select a VL endemic region that was conducive for field research. Based on our selection criteria (see Methodology), we selected western Kenya and northeastern Uganda for our field research.

### 3. Field Research With Direct Observations and Interviews With Stakeholders

**FIGURE 4.**
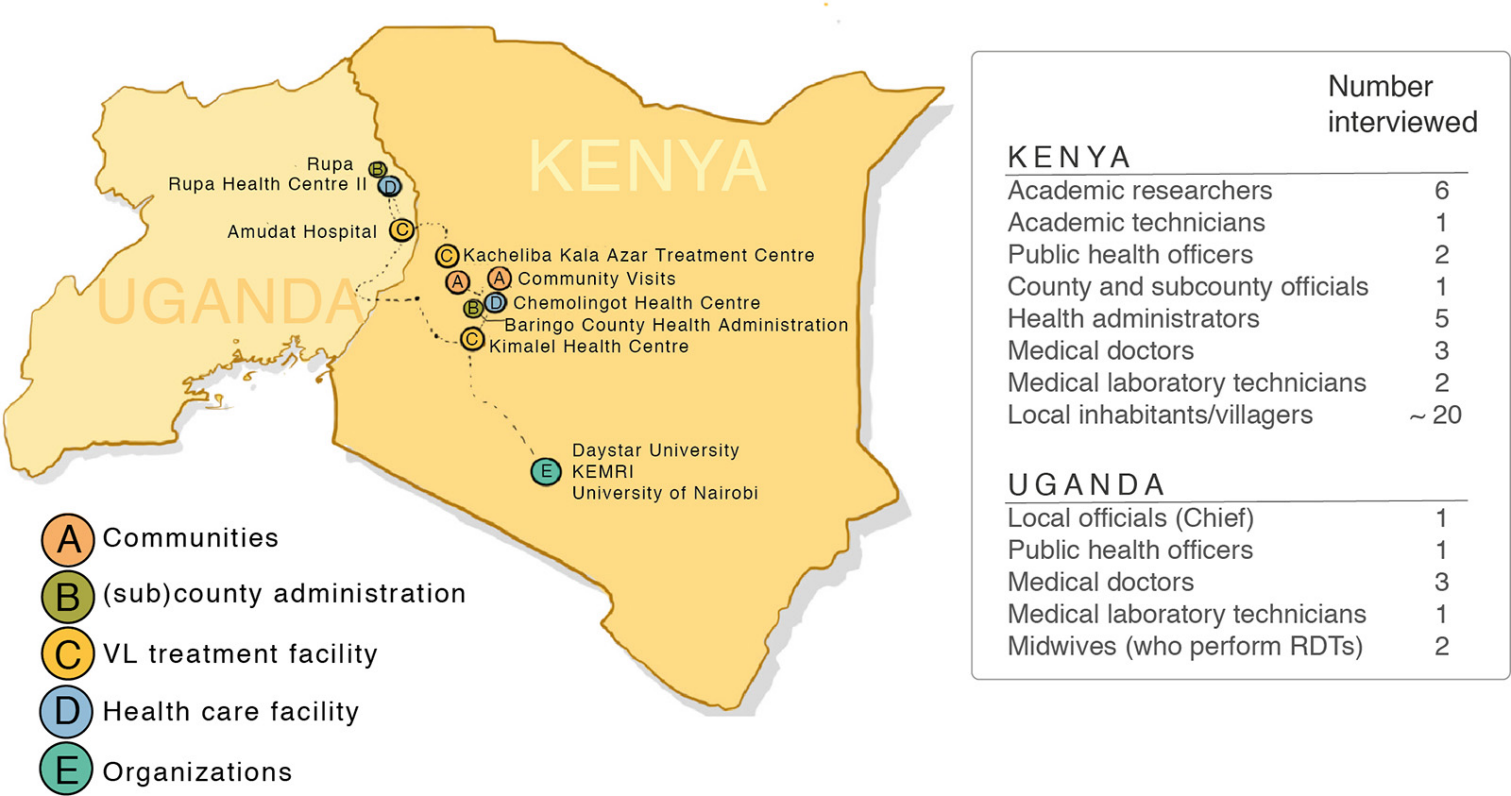
Number of Interviews Conducted with Stakeholders on Diagnosing VL in Western Kenya and Northeastern Uganda Abbreviations: RDT, rapid diagnostic test; VL, visceral leishmaniasis.

During our field trip to western Kenya and northeastern Uganda, we visited several Pokot tribal communities, health care facilities, and local organizations over 2 weeks in November 2018 (Figure 4). Our international team consisted of scientific re-searchers including a principal investigator, a postdoctoral researcher, and a PhD researcher, who are working together to develop innovative POC diagnostic tests for infectious diseases, and an industrial-design master student. Our local Kenyan team consisted of a public health officer, a research technician, and a research assistant. We engaged with county and subcounty officials, as well as with local health administrators and community health volunteers (CHVs). Our Ugandan team consisted of a medical doctor and a community health worker (CHW), and we engaged with key stakeholders such as the local chief in the Moroto district. A significant number of interviews were conducted as we followed the recommendations and advice of the locals about whom to speak to (Figure 4).

In Kenya, VL testing and treatment can be accessed at the local health care facilities that are located in the Rift Valley region: the Kimalel health center and a newly constructed treatment facility at the Chemolingot subcounty hospital (both within the Baringo County), and the Kacheliba health center (within the West Pokot County). In Uganda, the Amudat hospital is the only VL treatment facility. VL testing can be accessed in Rupa subcounty and by a mobile CHW in the surrounding regions, and patients are referred to the Amudat hospital for further testing and treatment.

### 4. Create Gigamaps and Patient Journeys Based on Insights Gathered

**FIGURE 5.**
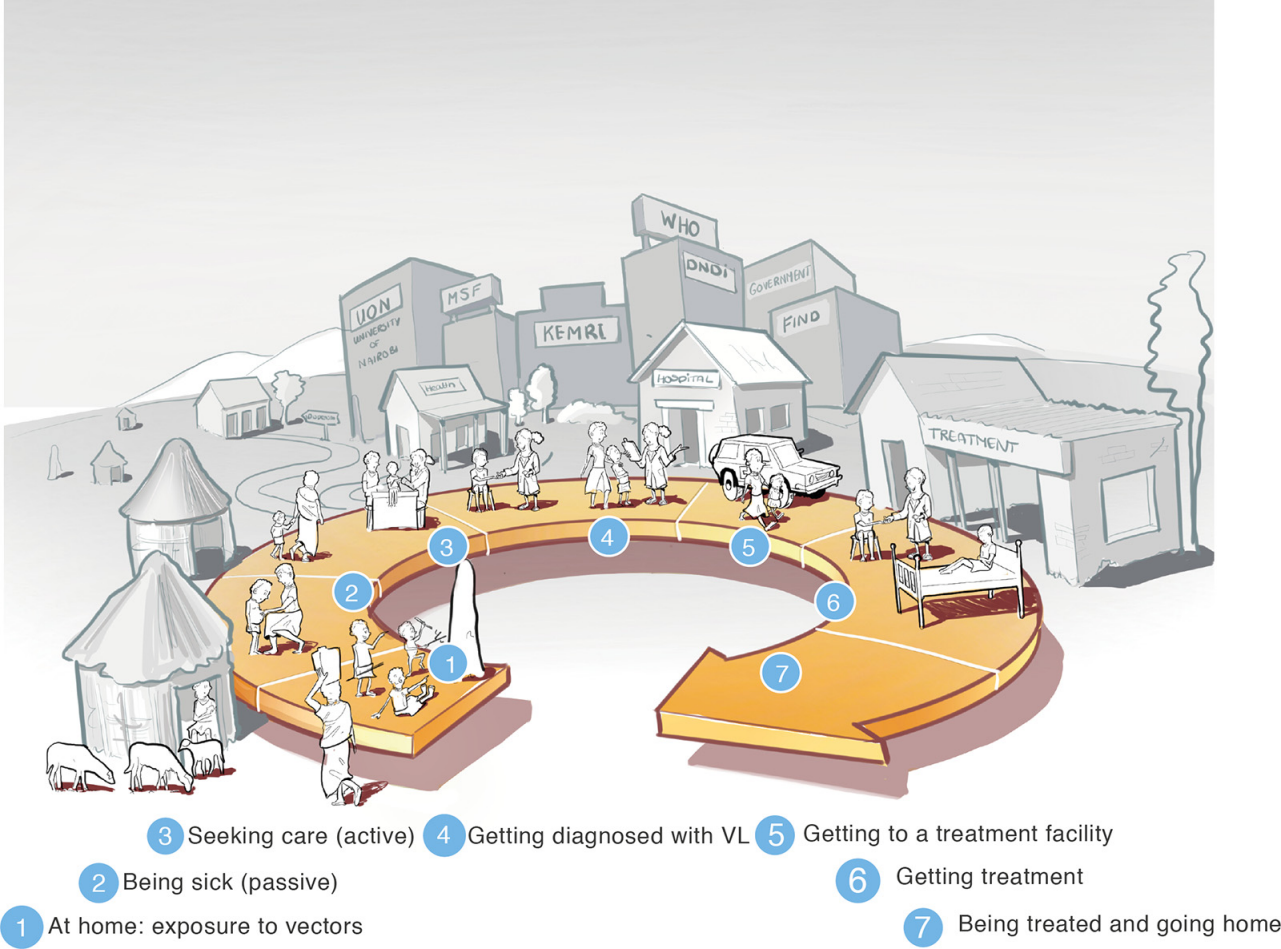
Gigamap Diagram Showing the Journey of a Patient Who Has Visceral Leishmaniasis From Infection to Treatment in Western Kenya and Northeastern Uganda Abbreviation: VL, visceral leishmaniasis.

A Gigamap of the health care system in western Kenya and northeastern Uganda was created based on the literature as well as on the insights obtained in the field. Seven phases were identified (Figure 5), from exposure to vectors at home (phase 1); being sick (passive) (phase 2); seeking care (active) (phase 3); getting diagnosed with VL (phase 4); getting to a treatment facility (phase 5); getting treatment (phase 6); and finally, to being treated and going home (phase 7). The Gigamap visualized the journey through the different levels of the health care system, starting from the rural setting (close to homesteads) and advancing towards more urban settings for treatment. The Gigamap also visualized the multiple stakeholders that are involved in the VL health care system.

**FIGURE 6.**
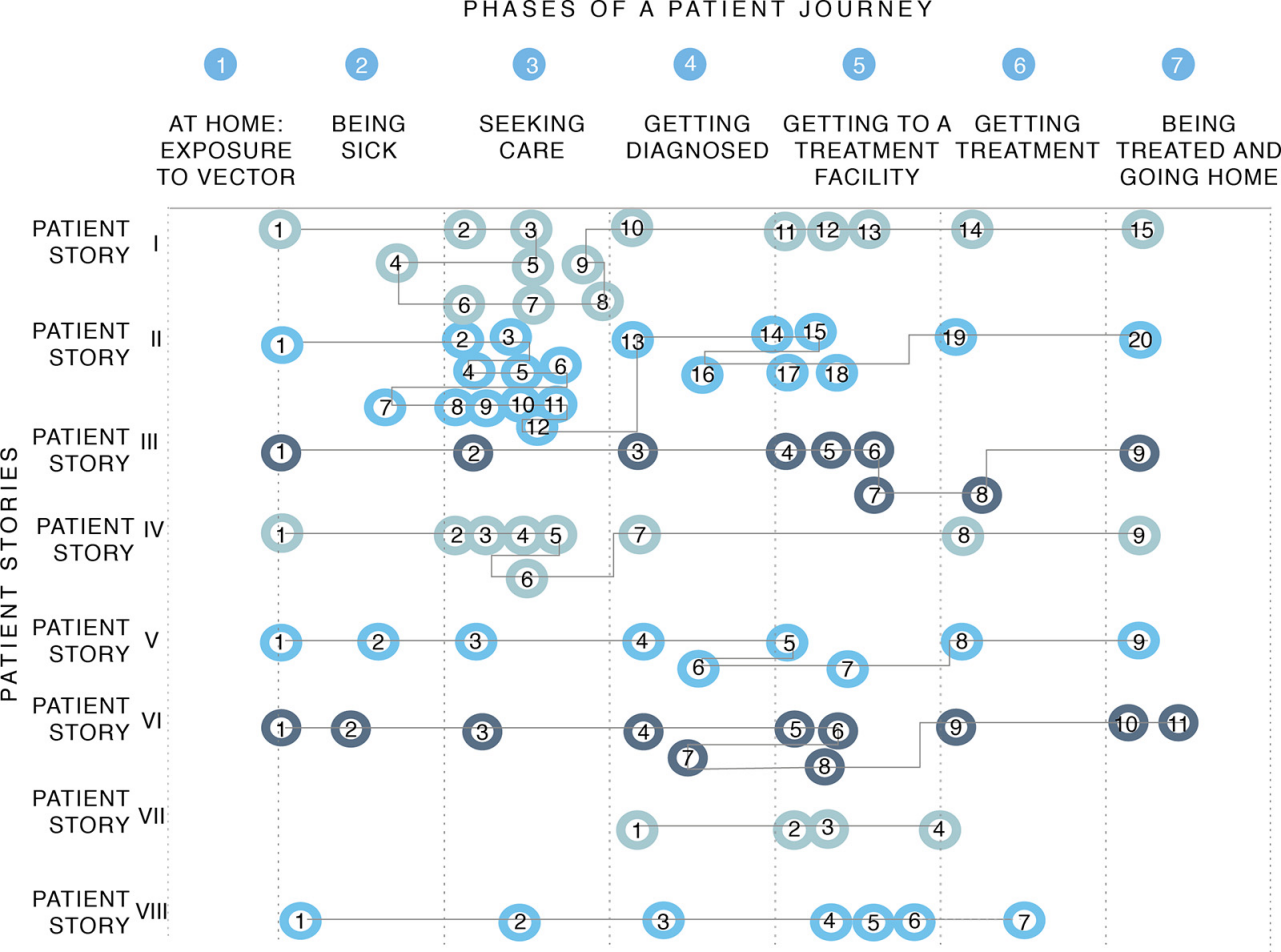
Phases of Patient Journeys That Represent the Number of Steps That Patients With Visceral Leishmaniasis Take From Infection to Treatment

Next, we created patient journeys (Figure 6). We related these journeys to the 7 phases defined in the Gigamap, to obtain a detailed overview of the challenges faced by a patient while seeking effective diagnoses and subsequent treatment. The 8 patient journeys (numbered as I to VIII) were a result of the information gathered in the field, and they represent a sequence of interactions between the patient, the health care system, and the stakeholders involved. A detailed description of patient story II can be found in the Supplement. Possible factors and barriers encountered during the patient journeys were identified. Several factors determined the progress of a patient through these different phases resulting in the least efficient (story II) and the most efficient (story VIII) journey from infection to the treatment.

An important observation during our study was the key role of the CHV/CHW as the closest link to the people in rural communities. In Kenya, CHVs and CHWs are trained on a variety of health issues including case definition, prevention, and control of common ailments, as well as nutrition and family planning. They facilitate access to health services through advocacy, outreach, referral, community education, informal mentoring, and social support.^39^ Thus, their role is to identify patients at the homesteads (small clusters of homes) (Figure 6, phases 1 and 2) and refer them to the local health care facilities for further diagnoses (Figure 6, phase 4). In Uganda, we observed that CHWs were trained to perform VL diagnostics in the field using the rK39 RDT. Due to ongoing VL clinical trials, which raises funding availability, CHWs in Uganda also had access to a motorcycle to allow them to reach secluded homesteads.

In contrast, due to limited clinical trials and a subsequent lack of funding, we observed that CHVs in Kenya are involved in identification of patients and referring them to local health care facilities. CHVs are well-respected members of the communities, such as teachers and ministers, and are trusted by the locals. Furthermore, due to language barriers between the locals and researchers from abroad, CHWs and CHVs played a key role in most interactions with the locals, patients, and health professionals.

The distance to a health care facility for diagnoses (Figure 6, phase 4) and treatment (Figure 6, phase 6) was found to be the most significant barrier that patients face when seeking health care. Diagnosis may take place at the homesteads by a CHW/CHV or at a health care facility, while treatment would take place at a higher-level facility such as a local hospital. Once diagnosed, either at the homesteads by a CHW/CHV or at a health care facility, patients in the East Pokot subcounty in Kenya that may have VL based on the rK39 RDT, currently travel approximately 80 km to the Kimalel hospital for another (confirmation) diagnostic test and for treatment, if needed. Due to the toxicity and costs of the treatment, patients are diagnosed multiple times to ensure that treatment is only prescribed when absolutely necessary (in contrast to antimalarial treatment that is prescribed more readily). The Chemolingot subcounty hospital treatment facility in Kenya is currently improving accessibility to treatment for patients in the East Pokot subcounty. However, in Uganda, patients from across the country need to travel to Amudat for VL treatment. Interestingly, many Turkanas in Kenya travel approximately 100 km to the Amudat hospital to seek treatment due to conflicts between their tribe and the Pokot in Kenya.

The distance to a health care facility for diagnoses and treatment is the most significant barrier that patients face when seeking health care.

Overlapping symptoms with other febrile illnesses often lead to misdiagnoses based on empirical clinical observations (signs and symptoms) (Figure 6, phases 2–7). In VL endemic regions, acute fever is often associated with malaria and other prevalent tropical diseases. Malaria RDTs and treatment are generally readily available, which encourages their use. Furthermore, a lack of VL awareness, even at the health care centers, promotes such misdiagnoses. Health education influences the ability of patients and health care workers to recognize VL. Increased VL awareness will have a positive influence on the journey of the VL patient because it increases the likelihood of recognizing VL at an early stage. Furthermore, we learned that children play in the termite mounds that are preferred resting and breeding sites for sandflies (the VL vector), and males sleep outside at night, which further exposes them to sandfly bites. Important risk factors include area ecology (humidity, heat), vegetation (acacia trees), livelihoods (pastoralism and proximity to livestock), and general behavior (outdoor sleeping).

A significant lack of resources is apparent for the diagnosis and treatment of VL, particularly in terms of health care personnel, diagnostic testing, and financial resources. Despite traveling far distances, patients have no guarantee that diagnostic procedures will be practiced immediately once they arrive at a health care facility for diagnoses. We learned that technicians generally close their laboratories for a day or two when they need to be in the field or are receiving training. Due to a general shortage of staff, diagnostic procedures are often delayed. Furthermore, RDTs such as the rK39 for VL are not always available, which promotes misdiagnosis of VL. Midwives at the Rupa health center in the Moroto district in Uganda, who routinely perform RDTs for malaria and other illnesses, informed us that the supply of RDTs for VL is not consistent. Thus, symptomatic patients that do not respond to malaria medication are then referred to the Amudat hospital (120 km) for diagnoses and treatment. Finally, the cost of traveling to a health care facility is a significant barrier, particularly when patients are asymptomatic and/or are simply not convinced that they are sick due to a lack of awareness. In general, a lack of financial resources contributes to poor health-seeking behavior (Figure 6, phase 2).

A significant lack of resources is apparent for the diagnosis and treatment of VL, particularly in terms of health care personnel, diagnostic testing, and financial resources.

We learned that many VL patients know that they are sick (Figure 6, phase 2), but do not actively seek health care (testing and treatment) due to the inaccessibility of health care facilities and the fear of encountering other tribes that may be hostile. We also learned that the Pokot and Turkana tribes in Kenya and Uganda are often in conflict, which makes it unsafe for members of either tribe to travel freely to seek health care. Such tribal conflicts inhibit patients from seeking health care and force them to travel to more distant health care facilities that are located away from a conflict zone. Such delays in seeking health care could further worsen the patient’s health, and they are often severely anemic and weak by the time they reach a health care facility. Furthermore, patients require screening for multiple infections, including VL, malaria, and tuberculosis, which is challenging when the patients are extremely weak. Stabilizing patients before starting them on VL treatment is also challenging because they may require blood transfusions and treatment for comorbidities, such as malaria, which require urgent attention before they can start VL treatment.

We observed that in general, VL patients also seek care from a traditional healer before considering visiting a health care facility because traditional healers are trusted, located closer to the homesteads, and often alleviate some of the initial symptoms. These deferrals worsen the patient’s health, thereby making the treatment more difficult. Unfortunately, critically ill patients often do not respond to the VL treatment, resulting in death. Such cases further strengthen the traditional beliefs and set a negative impression of health care facilities in the minds of health care seekers. Due to financial constraints and the cultural beliefs of the local population,^40^ the health care facility usually needs to arrange the last rites because the family does not come to the hospital to take responsibility for the deceased.

We observed a traditional patriarchal society, in which males leave the homesteads for work, and females take care of the homes and the children. For males, the loss of income due to the time that is spent traveling to seek health care contributes to poor health-seeking behavior (Figure 6, phase 2). For females, being unable to leave children unattended at home contributes to poor health-seeking behavior. We learned that women from the Pokot tribes often need permission from their husbands to leave their homes before taking themselves or a child to a health care facility. Thus, unequal decision-making power in the household contributes further toward delayed diagnoses and VL treatment for women and children.

Given all these factors, the total number of steps a patient takes to seek treatment can vary significantly. For example, seeking care in phase 3 requires many steps in complicated patient journeys, such as for patient stories I and II (Figure 6). This may be due to several of the aforementioned factors. For example, a lack of resources in the unavailability of staff or RDTs may result in the patient returning home (Figure 6, phase 2) without receiving a diagnosis, and later traveling back to seek care (Figure 6, phase 3). Traveling between phases 1 and 2 may be further hindered by other factors such as distance between the homesteads and the health care facilities or a lack of financial resources. Conversely, a patient journey may be as simple as patient story III whereby a patient progresses easily from being sick (Figure 6, phase 2) to being treated and going home (Figure 6, phase 7).

### 5. Create Use Case Scenarios

**FIGURE 7.**
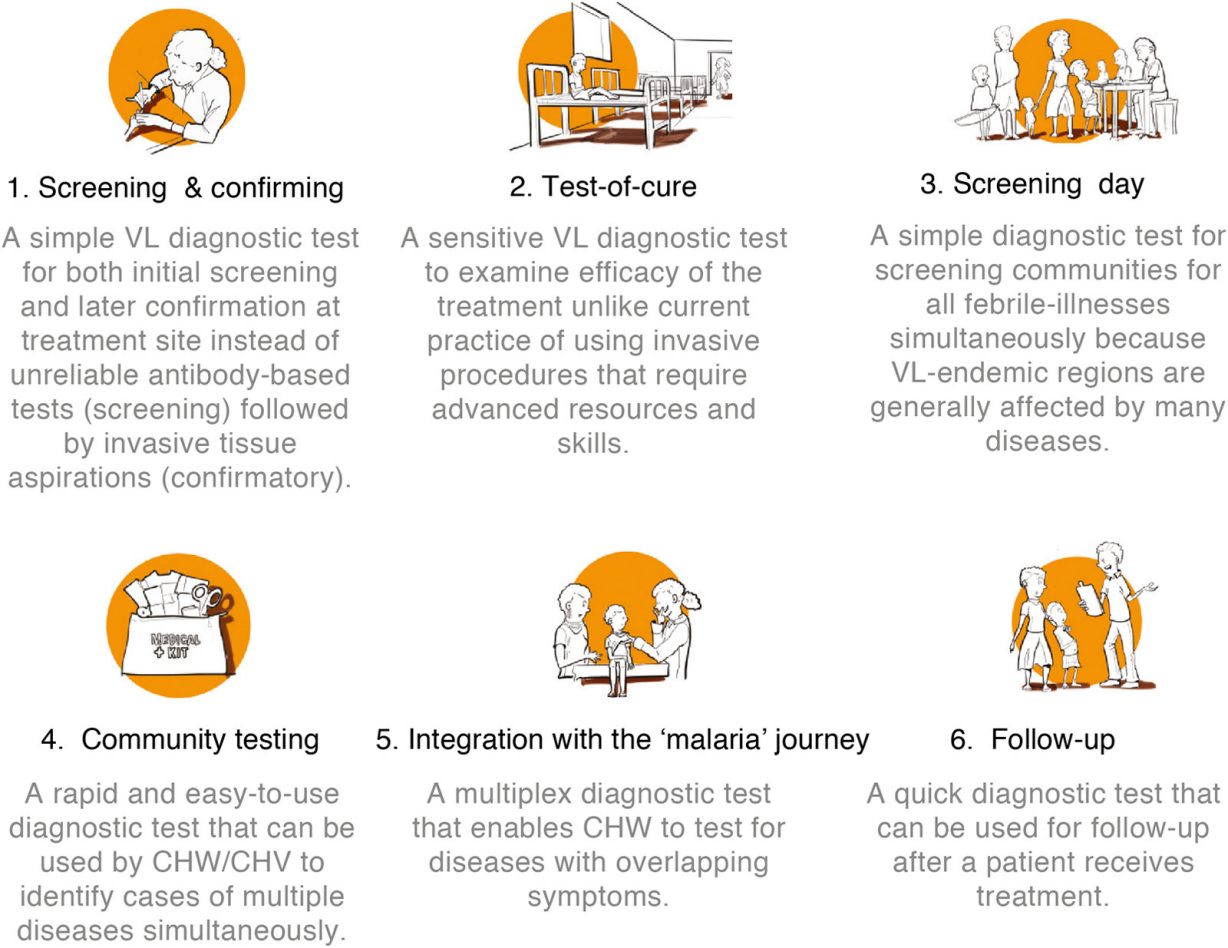
Six Scenarios That Elaborate on the Scope of Application of a New Point-of-Care Diagnostic Test Abbreviations: CHV, community health volunteer; CHW, community health worker; VL, visceral leishmaniasis.

Prompted by our extensive methodology, whereby we mapped the health care system in a specific endemic region and analyzed patient journeys, we sketched 6 use case scenarios based on the scope of application of a new POC diagnostic test (Figure 7). These different scenarios are based on considerations involving 3 elements: the characteristics of the new diagnostic technology, the contextual fit, and the local need. Each of the scenarios describes and visualizes a potential specific health care context in which a test based on a new diagnostic technology could be of added value to improve diagnostics in the health care system. Thus, each scenario represents a potential diagnostic setting.

### 6. Validation of the Use Case Scenarios

The aforementioned 6 scenarios were discussed with experts in the field, including a Kenyan public health officer and VL specialists at Médecins Sans Frontières, to identify the most urgent need based on the perspective of health care providers. Thereafter, we chose a researcher-centric approach to obtain a context-specific diagnostic need that would facilitate researchers to outline the technological requirements of a new POC test. Interestingly, 2 scenarios—screening and confirmation and a test-of-cure—were consistently identified as a priority for developing a CTPP, and we did not obtain any discrepancies in the opinions of the experts. The selection of these 2 scenarios was based on the following criteria: how well the scenario represents a diagnostic setting in the current health care context of VL; how well the scenario meets a local need in terms of VL case management; and how feasible it would be to implement a new POC test in the scenario.

Interestingly, 2 scenarios—screening and confirmation and a test-of-cure—were consistently identified as a priority for developing a CTPP.

The first scenario selected, “screening and confirmation,” is beneficial for a number of reasons. Given the poor performance of the serological rK39 RDT in east African countries, a more specific and more sensitive POC diagnostic test that can be implemented by end users with minimal training at the lowest level of the health care system is clearly required. Furthermore, it is crucial to consider the end user(s) of a POC diagnostic test because the level of training and availability of resources will influence the diagnostic setting. Thus, a simple, noninvasive, yet effective VL POC diagnostic test that can be used for initial screening and confirmation (scenario 1) would significantly improve VL case management because it would replace the rK39 RDT for initial screening and invasive splenic aspirations for confirmation. The added value of an effective screening and confirmation test is that patients could be screened more reliably at the lowest level of the health care system, for example, by a CHV/CHW who is closer to the homesteads, which would prevent patients from traveling unnecessarily to regional health care facilities.

The second scenario, “test-of-cure,” is useful because it would replace cumbersome procedures (i.e., microscopic analysis of invasively obtained splenic aspirations or molecular tests such as polymerase chain reaction) that are currently used for test-of-cure. Serological tests cannot serve as test-of-cure owing to persisting antibodies after VL treatment. Thus, a POC diagnostic test that can serve as a test-of-cure (scenario 2) would significantly improve VL case management by replacing invasively obtained splenic aspirations. The added value of a test-of-cure is that relapse of the disease, which occurs in approximately 10% of VL patients, could be detected at an early stage after treatment. Therefore, the test-of-cure scenario fills a critical gap in VL case management.

Scenarios 3 to 6 were excluded from further development of a CTPP. A screening day, as depicted in scenario 3, would be challenging to implement because one cannot ethically test everyone in a community, especially not asymptomatic patients, while symptomatic patients are covered in scenario 1. Additionally, it is difficult to get people to travel to a central location for a screening day because financial constraints or daily routines of herding cattle or taking care of the homesteads often restrict travel. Community testing, as depicted in scenario 4, is dependent on a multiplexed POC test that tests for multiple diseases simultaneously. Multiplexed tests are often more expensive and very few, if any, have been routinely used in the field.

Similarly, integration with the malaria journey, as depicted in scenario 5, is also dependent on a multiplexed POC test for VL and malaria. A well-known multiplex test from DIAMED for VL and malaria is relatively expensive and not routinely used in Kenya and Uganda. By contrast, malaria RDTs are affordable and are routinely used in the field. A follow-up test, as depicted in scenario 6, would be challenging to implement due to the nomadic nature of the inhabitants that we encountered.

A unidirectional problem-solving approach, instead of an interactive design-thinking approach that requires multiple iterations, would have yielded possibly 1 or 2 predictable scenarios instead of the detailed thorough 8 scenarios that were obtained in this study. Thus, after multiple iterations with a Kenyan public health officer and Médecins Sans Frontières, we concluded that scenarios 1 and 2, a test for screening and confirmation and a test-of-cure, are pivotal for VL management as they meet the local need and are feasible to implement. Furthermore, the CTPP developed for the 2 selected scenarios could be broadly applicable to the other 4 scenarios.

The CTPP developed for screening and confirmation and a test-of-cure could be broadly applicable to other scenarios.

### 7. Define the CTPP

Within each of these 2 selected scenarios, we identified variables that clarify how the diagnostic setting influences the features of the diagnostic test.

**FIGURE 8.**
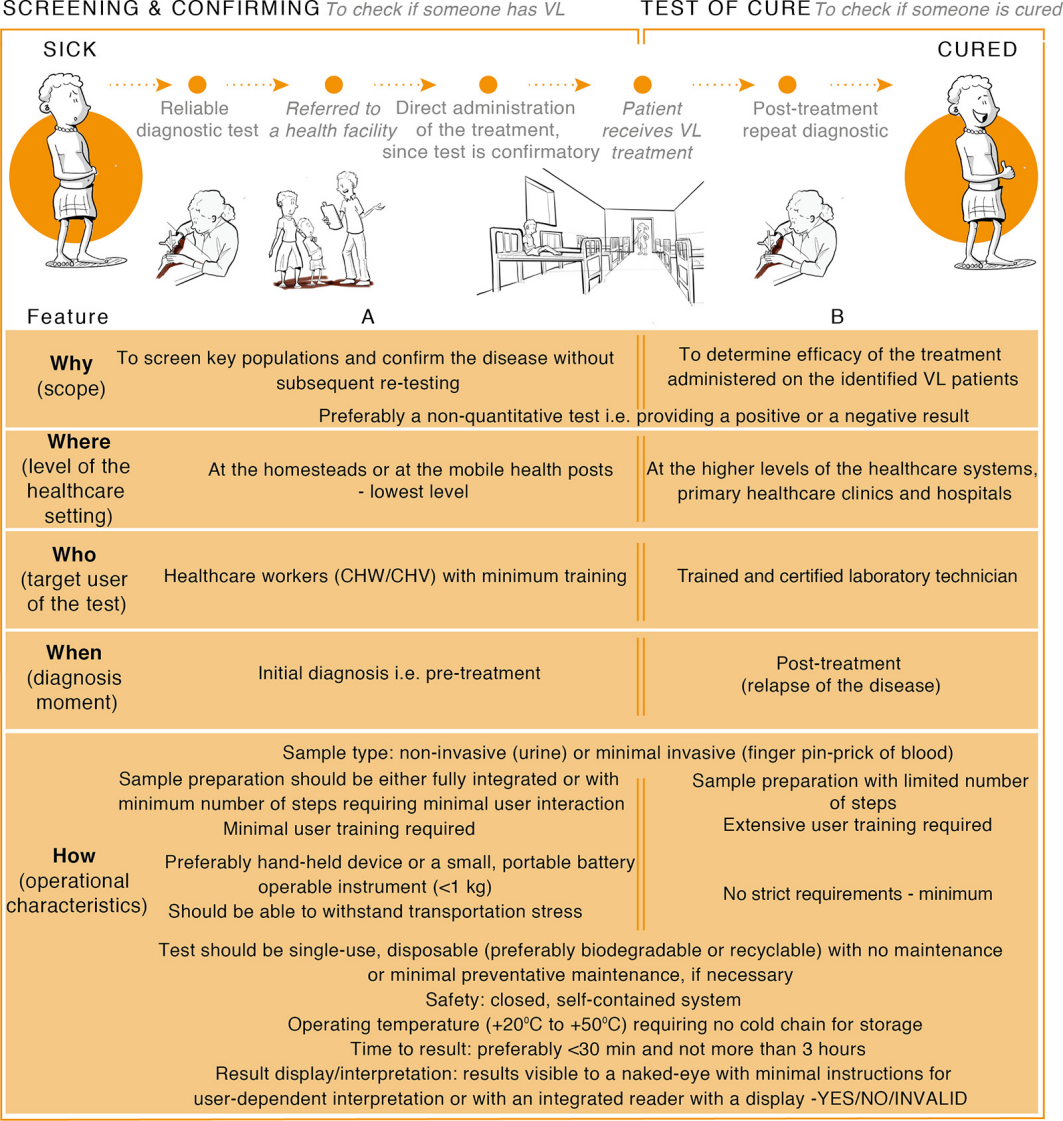
(A) CTPP for a VL Point-of-Care Diagnostic Screening-and-Confirmation Test, (B) CTPP for a VL Point-of-Care Diagnostic Test of Cure Abbreviations: CHV, community health volunteer; CHW, community health worker; CTPP, concept target product profile; VL, visceral leishmaniasis.

A CTPP such as formulated in Figure 8 presents the key features of a product that would fit a particular local health care context. The key features that a CTPP takes into account include variables such as why a test is performed (scope); where the test will take place (geographical location); which level of the health care system (such as at a home or a hospital); who the end user is (target user of the test); when the test will take place in the patients’ health-seeking pathway (diagnostic moment); and how the test will be conducted (operational characteristics of the test). In this study, the product is a new POC diagnostic test for VL in rural Kenya and Uganda. A CTPP is an efficient mapping tool that can guide the R&D of a new POC diagnostic test by determining the local needs, the end user(s), the intended use, and the adequate features of the test. We conclude that the approach presented in this study led to the development of a CTPP wherein the essential features of a POC diagnostic test for VL in Kenya and Uganda were identified more quickly compared with a TPP. The added value of a CTPP is that it does not impose overly stringent guidelines on the researcher during the early stages of R&D. Instead, a CTPP aims to guide the research and not limit the potential of the new POC diagnostic test that is under development.

The approach presented here led to the development of a CTPP wherein the essential features of a POC diagnostic test for VL in Kenya and Uganda were quickly identified.

## DISCUSSION

The global diagnostic need for NTDs demands innovative solutions that are customized to the local health care context. To develop a technology that can be implemented in the relevant context, researchers must get access to a comprehensive, yet easily accessible overview of the status quo to understand the challenges and limitations of the existing health care system. Despite extensive literature reviews on VL diagnostics, treatment, and management, an evident knowledge gap persists, which prompted us to conduct field research in VL endemic regions and develop the notion of a CTPP.

Although it is imperative to conduct a comprehensive context analysis before and during R&D of the technology, this is often done only at a much later stage by specialized teams, which may not include scientific researchers. The present study was conducted by scientific researchers who are developing a POC diagnostic test in collaboration with industrial-design experts to define scientifically feasible innovative solutions for VL diagnosis in a resource-limited setting. In contrast to a TPP that comprises a much larger group of experts such as social scientists and policymakers, a CTPP can be formulated effectively in a fast manner with a team that includes technical researchers, health care experts, and industrial designers.

Initially, it appeared challenging to determine how to effectively engage with local stakeholders during a limited time in the field. From the very beginning of our approach, we overcame many logistical and cultural barriers by engaging with local stakeholders. Apart from engaging with medical professionals (doctors, nurses, and laboratory technicians), we spent valuable time with the CHWs/CHVs and observed their crucial role in VL diagnosis in the field. We observed many similarities in the health care systems between Kenya and Uganda, which is unsurprising given the common border and a shared history; therefore, we created a combined Gigamap of their VL health care systems. We observed many challenges that are encountered by the locals: food and water insecurity, which causes malnutrition; remoteness; and a lack of infrastructure and poor health care systems, which collectively adversely affect access to adequate diagnoses and treatment. Furthermore, the lack of resources, both financial and in the availability of health care staff and RDTs for VL, and poor health-seeking behavior, which is exacerbated by the lack of education that is prevalent in the remote regions, impede effective VL management.

Conducting field research provided rich sources of information for understanding how new POC diagnostic tests can fit into a specific health care context. We would like to highlight the importance of international cocreation through active collaborations between all stakeholders, including academia, industry, nonprofit, and governmental organizations.^9^ Cocreation with local experts is necessary to understand the implementation need for a new POC diagnostic test, as well as to ensure the sustainable use of the POC test in the field by building trust and mutual interest and creating a foundation for knowledge transfer to engage locals in the future. New technologies are often mistrusted by health care providers, especially in remote settings, due to a lack of understanding of the complex research behind the development of the new technologies. Thus, cocreation with the local stakeholders starting from the design phase (conceptualization) to the prototype phase (realization) ensures a strong relationship with the end users. The approach that we adopted, in fact, promoted collaboration (and not competition) between the key stakeholders.

A number of key findings that were learned from the approach are as follows:

- A CTPP is an effective new tool that can aid R&D researchers in matching the technology that they develop to a specific health care context more quickly than a conventional target product profile.
- The role of local volunteers and community health care workers is critically important for access to diagnostics in resource-limited settings. With improved yet simplified VL POC diagnostic tests, CHWs/CHVs could perform diagnosis of VL closer to the homesteads.
- A noninvasive test-of-cure and a screening and confirmation test will significantly improve the management of VL in the endemic regions. This would greatly benefit patients, particularly immunocompromised patients who are at a higher risk of relapse, as well as help pharmaceutical researchers and clinicians who are developing and testing new VL treatment regimens.
- The cost of the diagnostic test is an important factor to consider, especially in view of the fact diagnoses needs to be repeated, to screen the patient in the field initially, and again at the treatment facility to rule out any procedural error and to justify the toxicity and expense of VL treatment. Thus, the diagnostic test needs to be affordable.

Key implications learned from the approach are:

- Early during the R&D stage, researchers should consider who will administer the test (patient, health care worker, doctor) and for what purpose.
- Program managers should consider that the training level of staff and volunteers and the availability of the resources are the critical determinants for using a diagnostic test.
- Researchers should consider that introducing themselves to local communities and stakeholders early will improve the willingness of the communities to implement the new technology. Upon further development, testing prototype devices can be facilitated by the local East African partners, thus strengthening international cocreation, and increasing the probability of success of a new POC diagnostic test beyond a mere proof-of-principle.

The plethora of information gathered in the field was comprehensively processed using our methodology, which includes design-thinking tools such as visual thinking, leading to the development of a Gigamap, patient journeys, and the consequent use case scenarios that are presented in this study. The visual thinking was used as a means to summarize, validate, and communicate key insights from the field research to the stakeholders, as well as to create an aligned vision within the team. The visualizations allowed us to identify where, when, and how a new POC diagnostic test can fit into the health care system within a resource-limited endemic region, in the form of a CTPP, which is an efficient mapping tool compared with a TPP. A CTPP approach was applied to sufficiently scope the problem of VL diagnostics, gather contextual information, and define the adequate features of a new POC diagnostic test.

## CONCLUSION

Disease eradication requires improved diagnostic tests, as well as an efficient system to successfully deliver and implement them in the appropriate settings.^41^ As this is largely dependent on the local capacity and the willingness of key stakeholders to participate, solely designing a POC test for a particular setting does not ensure successful implementation of the test.^5^ Designing a product for the end user is complex since a wide range of political, social, cultural, and environmental factors contribute, but it is worth the added time, effort, and resources to realize a successful development and implementation of a new POC diagnostic test.

In this article, we presented an approach that included design-thinking principles to formulate a CTPP that consists of multiple steps. Our approach moved from identifying gaps in current VL diagnosis in endemic regions by critically reviewing the existing literature, to selecting an endemic region to validate the literature findings and conduct direct observations in the field. After that, we used visual thinking to create Gigamaps and patient journeys based on the combined insights that were obtained from the literature and the field research, which led to valuable use case scenarios that describe the ideal setting for a new POC diagnostic test. Finally, we used these collective data to formulate a CTPP for a new POC diagnostic test that is specific for VL diagnostics in resource-limited settings.

In summary, we introduced the notion of a CTPP as an effective toolbox to match the development of a POC diagnostic test and the health care context for its application. More generally, we anticipate that a CTPP will be a useful new tool that enables researchers to match the development of new diagnostic tests or medical equipment, and the local health care context in which they will be used. We envision that a CTPP will enable researchers to ruminate on the new product and facilitate the iterative design process—and ultimately benefit global health.

## Supplementary Material

20-00028-Diehl-Supplement-clean.docx

## Notes

**Peer Reviewed**
