# Supplementary material for: Matching Development of Point-of-Care Diagnostic Tests to the Local Context: A Case Study of Visceral Leishmaniasis in Kenya and Uganda
Source: Glob Health Sci Pract. 2020 Sep 30;8(3):549–65. doi: 10.9745/GHSP-D-20-00028 (PMC7541118; doi:10.9745/GHSP-D-20-00028)
Supplement: 20-00028-Diehl-Supplement-clean.docx [file 20-00028-Diehl-Supplement-clean.docx]

**Supplement**

**Patient story II – A 5-year-old who was referred multiple times**

1. A 5-year-old girl does not feel well. She has a fever and general malaise.
2. Her mother assumes that she has malaria and goes to the nearest health care facility to get malaria medication that is readily available.
3. The 5-year-old girl takes the malaria medication but does not feel better.
4. Her mother asks their neighbor for advice but she also does not know what is causing the illness. Weeks pass by.
5. The 5-year-old girl gets even more sick. Her spleen starts to swell. Her mother decides to take her back to the health care facility.
6. At the health care facility, they test the 5-year-old girl for malaria. The test results are negative. However, they are sure it is malaria because her symptoms are the same as malaria symptoms. They prescribe another dose of malaria medication and send the girl home.
7. The 5-year-old girl does not get better. She gets progressively more ill.
8. Her mother takes her back to the health care facility and asks for help because she is convinced now that the 5-year-old girl does not have malaria.
9. The nurse agrees that it cannot be malaria and tests the 5-year-old girl for other illness such as tuberculosis. However, all the test results are negative.
10. The 5-year-old girl is then referred to another health care facility. The mother and child walk 80 km to the next health care facility.
11. At the health care facility, another nurse tests the 5-year-old girl for several illness including malaria. The nurse suspects that the 5-year-old girl might have visceral leishmaniasis (VL) but she does not have any VL tests. The 5-year-old girl is then referred to the nearest hospital.
12. The mother carries the child to the nearest hospital which is 20 km away. This journey takes many hours.
13. The 5-year-old girl is tested positive for VL with the rK39 rapid diagnostic test (RDT). She is referred to another hospital that also has a VL treatment center.
14. The staff call the VL treatment center and request a vehicle to transport the 5-year-old girl—without her mother who has to return home.
15. The vehicle transports the 5-year-old girl to the treatment center.
16. When the 5-year-old girl arrives at the treatment center, she is tested again for VL with the rK39 RDT. The result is again positive. She then gets further blood tests done for liver function and anemia, to name a few. These tests are required before VL treatment will be administered.
17. The blood tests reveal that the 5-year-old girl’s hemoglobin levels are too low for treatment (less than 4.0 g/dL).
18. The 5-year-old girl is then taken to another treatment center for a blood transfusion before she can start VL treatment. This takes 1 week.
19. After 1 week, the 5-year-old girl is taken back to the treatment center and starts VL treatment.
20. After 17 days of VL treatment, she is taken back to her family, treated and feeling well.
